# Fundamentals, real-time uncertainty and CDS index spreads

**DOI:** 10.1007/s11156-023-01127-6

**Published:** 2023-04-12

**Authors:** Alena Audzeyeva, Xu Wang

**Affiliations:** 1 Keele Business School, Keele University, Staffordshire, ST5 5BG UK; 2 Faculty of Business and Law, University of Northampton, Northampton, NN1 5PH UK

**Keywords:** CDS index, Credit spreads, Macroeconomic fundamentals, Macroeconomic uncertainty, Economist survey nowcasts, Credit spread forecasting, E44, G12, G17

## Abstract

The high level of economic uncertainty linked to the pace of the recovery process can persist after a crisis and has implications for the market pricing of firms’ credit risk reflected in credit default swap (CDS) spreads. This paper examines the role of key proxies for the economic state and its real-time uncertainty in determining Northern American CDX index spreads. Focusing on the recovery period following the 2007–2009 global financial crisis, we find that measures of economic output, employment, inflation, and economic uncertainty, all significantly influence CDX spreads, beyond the impact of conventional determinants. Furthermore, our results provide evidence that the sensitivity of investment-grade and high-yield CDX differs across economic aspects. Moreover, our out-of-sample predictive analysis identifies indicators and uncertainty measures with significant predictive content for quarter-ahead CDX spreads. Taken together, our findings indicate that academic modelers and practitioners employing more accurate representations of the macroeconomy in CDS modeling and analysis can improve upon the models that rely solely on the typically employed economic output variables or on broad data aggregation.

## Introduction

The globally dominant credit derivative market, the CDS market, has undergone a sizable compression in the aftermath of the 2007–2009 global financial crisis (GFC) as market participants sought to mitigate the significant risks exposed by the crisis. However, the decline in activity mainly affected the single-name CDS market, with the liquidity of CDS index instruments remaining high (Lando 2020). Post-GFC events further highlight the increasing importance of CDS index instruments. During the post-crisis recovery period, the market risk transfer activity (MRTA) rose for CDS indices to reach $5.8 trillion in the second quarter of 2019, contrasting with the single-name CDS MRTA that leveled off at $0.6–0.7 trillion per quarter in the prior 13 quarters (ISDA 2019).^1^ The onset of the economic crisis triggered by the COVID-19 pandemic has further led to the 116% surge in the CDS traded notional value between September 2019 and March 2020, driven by market participants seeking protection against sharply elevating credit risk, with the rise largely accounted for by increased trading in CDS index instruments (Boyarchenko et al. 2020; Fekete and Janosik 2020).

The rapidly growing use of CDS indices for credit risk transfer by market participants points to the elevated importance of managing the economy-wide credit risk exposure as CDS indices offer credit protection on the portfolio of names included in the index, contrasting with a single entity in a single-name CDS, thus, allowing investors to efficiently implement a range of hedging and speculation strategies whereby they hedge against negative market-wide events or take views on either the entire market or a market sector captured by the index (Adam and Guettler 2015; Oehmke and Zawadowski 2017).^2^ However, since the state of the economy is not known with certainty when making trade decisions, assessing and managing the economy-wide credit risk exposure remains a nontrivial task (e.g., David 2008; Gilbert et al. 2017). The literature has documented that various available macroeconomic indicators can provide useful signals about the economic state and reveal more subtle information about specific economic drivers of asset prices (Gilbert 2011; Gilbert et al. 2017; Nadler and Schmidt 2016). Thus, market practitioners, economic policy makers, and financial regulators can gain from a better understanding of how developments in specific aspects of the economy influence CDS indices.

Consequently, our study systematically evaluates the role of key macroeconomic fundamentals along with associated real-time uncertainty in these fundamentals in determining CDS index spreads. We study the impact of variables spanning economic output, employment, and inflation aspects of the economy. Beyond analyzing contemporaneous influences, we assess the predictive content in macroeconomic fundamentals and uncertainty measures for future CDS. In doing so, we extend the analysis in previous CDS studies by focusing on the informational content for CDS in indicators reflecting specific economic aspects and associated uncertainty, contrasting with broad measures of the macroeconomy such as the GDP growth, indices aggregated across a broad range of economic indicators, or financial market measures, employed in previous CDS studies. We account for uncertainty in assessing the economic state by adopting a set of novel measures of economic uncertainty based on real-time subjective economists’ nowcasts of key macroeconomic indicators made prior to their release.

Inspired by Merton (1974) structural model of credit spreads and its extensions, the voluminous literature of CDS determinants has predominantly focused on the impact of firm-level characteristics, largely omitting macroeconomic influences from consideration. Notable exceptions include Tang and Yan (2010) and Baum and Wan (2010), however, they focus on single-name CDS as opposed to CDS indices and consider only few generic measures of macroeconomic conditions such as the GDP growth and related uncertainty.^3^ However, the long delays in the availability of GDP and the limited coverage of industrial production data they use limits the practical utility of their findings for market practitioners. In a contrasting study, Kim et al. (2017) employ financial market variables together with an inflation measure to capture the macroeconomy. Unfortunately, their approach prohibits identifying the impact of specific macroeconomic drivers. Moreover, market-data-based measures of economic conditions have been criticized for being extremely noisy (e.g., Claessens and Kose 2017). In another study, Galil et al. (2014) examine several competing CDS models, employing a model with macroeconomic determinants as one of the competitors. They find that even though their macroeconomic variables can explain some of the variation in CDS spread changes, they become insignificant once firm-specific and market variables are added to the regression. However, the findings for single-name CDS in these studies are not directly applicable to CDS indices (Alexander and Kaeck 2008; Wisniewski and Lambe 2015).

Our analysis focuses on North American CDX indices: the North American Investment-Grade CDX index (CDXIG) and the North American High-Yield CDX index (CDXHY), which jointly accounted for about half of the total global CDS index market activity in 2018–2019 (ISDA 2019). Employing data at a monthly frequency from the economic recovery period between July 2009 and December 2018, our analysis produces three major findings.^4^ First, it reveals that economic output, labor market conditions, inflation, and labor market-based measures of economic uncertainty, all provide significant additional explanatory power for both investment-grade and high-yield CDX spreads, beyond that of the conventional determinants informed by the structural models. The conventional variable set augmented by macroeconomic variables can explain close to 79% and 86% of the spread variation over time for CDXIG and CDXHY, respectively, helping address the “credit spread puzzle” that highlights the low explanatory power of the conventional determinants documented in the credit spread literature. Second, we find that CDXIG is more sensitive to measures of economic output than CDXHY while CDXHY exhibits a higher sensitivity to both labor market conditions and associated uncertainty. These findings caution against relying on economic output variables alone or a broad variable aggregation that are typically used to capture the macroeconomy in the CDS modeling and analysis. Third, our OOS predictive analysis further confirms that some fundamentals and uncertainty measures have significant predictive content for a-quarter-ahead CDX spreads, beyond that of the conventional determinants, which has important implications for market practitioners’ trading strategies.

Our analysis contributes to three strands of literature. First, it adds to the growing literature on determinants of CDS index spreads by Byström (2006), Alexander and Kaeck (2008), Breitenfellner and Wagner (2012), Chan and Marsden (2014), and Wisniewski and Lambe (2015). Only the latter two studies explicitly investigate macroeconomic influences. However, our study differs from theirs in two important ways. First, we examine the impact of specific economic drivers on CDS spreads. In contrast, Wisniewski and Lambe (2015) examine the role of *policy uncertainty* whereas Chan and Marsden (2014) study the *generic effect of the macroeconomy* on daily changes in CDS spreads by using an index of business climate along with related volatility, both based on data aggregated across a broad range of macroeconomic indicators. As such, Chan and Marsden (2014) assume that macroeconomic conditions can be adequately summarized by a broad index measure, effectively constraining the CDX response to being proportionate to underlying components; we relax that restriction in our study. Importantly, such broad measure together with their supplementary market-based measures, which themselves are subject to complex macroeconomic influences, don’t permit examining the impact of specific macroeconomic drivers on CDX, which our study addresses.^5^ Second, our analysis, using novel survey-based measures of economic uncertainty (a) overcomes many of the known biases of model-implied measures in Chan and Marsden (2014) as well as Baum and Wan (2010) and Tang and Yan (2010) focusing on single-name CDS, and (b) accounts for various sources of macroeconomic uncertainty, unlike generic or aggregate measures commonly used in the CDS literature.

Secondly, our analysis extends the empirical work of Benkert (2004), Alexander and Kaeck (2008), and Ericsson et al. (2009) among others who assess the ability of conventional theoretical determinants, central to the Merton (1974) structural model and its many extensions such as Longstaff and Schwartz (1995) and Anderson and Sundaresan (1996), to explain CDS spreads. We extend their work by augmenting the set of conventional determinants with a set of macroeconomic indicators and uncertainty measures, motivated by more recent structural models with macroeconomic influences such as Tang and Yan (2006) and David (2008).

Finally, we add to a nascent strand of literature that focuses on CDS predictability out-of-sample, or in real time. We build upon only two such studies: Narayan et al. (2014) explore the role of price discovery in CDS and equity markets for forecasting daily single-name U.S.-based CDS returns and Avino and Nneji (2014) contrast the predictive performance of linear regression models to Markov-switching models for European iTraxx index spreads during the 2007–2009 financial crisis, documenting a superior performance of linear models. We extend the OOS predictability evidence to CDX index spreads, omitted from previous studies, also expanding the set of candidate predictors therein to include macroeconomic variables. Moreover, we extend the evidence in Avino and Nneji (2014) on the CDS index predictability during the GFC by findings relating to the subsequent economic recovery period.^6^

The rest of the paper is set out as follows. Section 2 reviews the related literature and develops our tested hypotheses. Section 3 outlines our analytical approach, with Sect. 4 detailing the data. Sections 5 and 6 outline the empirical methodology and summarize the findings for the regression analysis and OOS predictive analysis, respectively. Section 7 concludes.

## Related literature and hypotheses

The structural model of Merton (1974) and its extensions such as Longstaff and Schwartz (1995), Collin-Dufresne et al. (2001), and Zhou (2001) link firms’ credit risk to the evolution of the firm’s asset value, identifying the asset value growth and volatility together with the risk-free interest rate as key drivers of credit spreads. Even though ample empirical evidence, for example, in Benkert (2004), Alexander and Kaeck (2008), Ericsson et al. (2009), and Breitenfellner and Wagner (2012), document the expected relationship between the theoretical determinants and credit spreads, the conventional structural models have had limited success in matching observed credit spreads, with this issue commonly referred to as “the credit spread puzzle”; see, for example, Elton et al. (2001) and Amato and Remolona (2003).

More recently, Tang and Yan (2006) and David (2008) proposed structural models that depart from the conventional approach by explicitly linking credit spreads to macroeconomic fundamentals and surrounding uncertainty. Tang and Yan (2006) consider the equilibrium in the macroeconomy that depends on macroeconomic conditions captured in their model by the output growth and volatility and aggregate risk aversion. The pricing kernel and the risk-free rate, jointly determined in equilibrium, are, in turn, utilized for pricing the firm’s debt and equity. In their model, higher output growth is associated with a higher drift of the firm’s cash flow process which increases the likelihood of debt repayment, thus, reducing the probability of default and lowering credit spreads. At the same time, an increase in uncertainty about output growth widens the risk premium embedded in credit spreads. Further empirical analysis in Baum and Wan (2010) and Tang and Yan (2010) support the model prediction about the effect of economic output growth and related uncertainty in the single-name CDS context.

However, David (2008) and David and Veronesi (2013) point out that market participants cannot observe the current economic state, which is hidden from them in real time. David (2008) addresses this issue by developing a generalized model exploiting Bayesian learning, in which investors learn about the economic state over time, with the expected state of the economy and surrounding uncertainty both influencing the firms’ solvency indicators and asset values that are endogenously determined. In the model, investors learn about the economic state by observing inflation and earnings growth. High inflation signals an increased likelihood of real earnings falling to a low growth state, causing credit spreads to widen, with uncertainty also affecting credit spreads. David (2008) shows that his model generates more realistic values of credit spreads than those produced by conventional structural models.

David and Veronesi (2013) further note that in practice investors utilize signals from various macroeconomic variables to form their view about the state of the economy. The informational content in the additional variables can be particularly important during periods of relatively low and stable inflation (e.g., Amato and Luisi 2006). Professional investors are known to monitor particularly closely labor market indicators, placing them among the three most important measures together with inflation and economic output. Consistent with this practice, studies of the effect of macroeconomic announcements identify employment variables as particularly influential for asset pricing. For instance, Nadler and Schmidt (2016), Gilbert et al. (2017), and Huang and Kong (2008) document a significant impact of labor market related news on asset prices in the contexts of U.S. equities, Treasuries, and corporate bonds, respectively. In line with the investor pricing behavior, Gilbert et al. (2017) find key employment indicators among those highly informative about the state of the economy. As these closely monitored employment indicators tend to be more flexible relative to economic output and inflation measures, which typically exhibit stronger persistence, they can supplement or modify signals about the trend in the economic recovery prospects contained in measures of economic output and inflation.

Taken together, these studies provide arguments for economic output, inflation, and employment measures, all being of relevance for CDS pricing. Also, uncertainty about these economic aspects is likely to inform investors’ views about the uncertainty surrounding the economic state, embedded in the CDX pricing. We formulate our first hypothesis accordingly.

### Hypothesis 1

CDX spreads are tighter when economic output growth is stronger (H1a), employment is stronger (H1b), inflation is lower (H1c), and macroeconomic uncertainty is lower (H1d).

Firm-specific determinants such as the asset value growth and volatility, which are central to the early structural models and widely employed in empirical CDS studies, are likely to absorb at least some of the macroeconomic influences, potentially serving as measures of macroeconomic risk factors in our CDS portfolio context. Using the joint structural-equilibrium modeling framework, Bhamra et al. (2010a, b) and Chen (2010) provide an underlying theoretical intuition. The two studies treat firms’ financing and default-related decisions as endogenous, both influenced by the state of the economy. The models account for the impact of macroeconomic conditions on default probabilities and credit spreads through modifying firms’ financing decisions determining firm-specific leverage and feeding into equity values and growth rates. Such indirect impact of macroeconomic conditions is empirically evidenced in Korajczyk and Levy (2003) who document that macroeconomic influences account for 12 to 51% of the time-series variation in firms’ leverage. In their analysis of the joint impact of market conditions and firm-specific variables, Tang and Yan (2010) further find that most of the macroeconomic impact on CDS spreads occurs indirectly, via its interaction with firm-specific characteristics.

Nevertheless, as macroeconomic conditions also influence aggregate investor risk aversion, modifying prices of risk and, hence, the risk premium embedded in CDX spreads, such direct market pricing channel remains of relevance for CDX, with the risk-free rate employed in conventional models alone unlikely to capture the influence. Collin-Dufresne et al. (2001), Blanco et al. (2005), and Ericsson et al. (2009) among others provide indirect evidence to that effect by documenting a sizable share of common variation in credit spreads and spread changes that cannot be explained by conventional theoretical determinants. In more recent analyses, Kim et al. (2017) and Chan and Marsden (2014) report the importance for CDS of the expected market risk premium and default risk premium, respectively, even after accounting for the effect of the conventional determinants, with both risk premia reflecting macroeconomic conditions. We formulate our hypothesis 2 accordingly.

### Hypothesis 2

Macroeconomic variables provide additional explanatory power for CDX spreads, beyond that of the conventional theoretical determinants.

Previous evidence suggests that firms of high and low credit quality differ in their exposure to macroeconomic conditions, with the differences likely to be reflected in the sensitivity to macroeconomic changes for high-yield and investment-grade CDX (e.g., Amato and Luisi 2006; Wu and Zhang 2008; Zhou 2014). However, systematic evidence is lacking in the CDS context. Moreover, few existing studies report conflicting results for economic regimes of relative stability, also analyzed here. For example, Kim et al. (2017) find that during such regimes, the impact of the business cycle on CDS spread changes is stronger for investment-grade CDS than high-yield CDS whereas Chan and Marsden (2014) document that their measure of business climate influences CDXHY but not CDXIG.^7^

A key reason, cited in the literature, for potential differences in the high and low-rated firms’ exposure to macroeconomic conditions is that firms of low credit quality tend to rely more on external financing, the availability and costs of which vary considerably over the business cycle (e.g., Diamond 1991, 1993; Campello et al. 2010; McLean and Zhao 2014). When macroeconomic conditions worsen or become more uncertain, external financing becomes constrained and costly, which primarily affects low-rated borrowers. Moreover, high adverse selection costs during bad times mean that low-rated firms can typically access only costly short-term debt financing, with this problem further exacerbated by refinancing risk and debt rollover risk, whereas high-rated firms tend to retain access to more favorable financing options (e.g., Diamond 1991, 1993; Datta et al. 2019; Liu et al. 2021). This evidence points to investment-grade borrowers being less exposed to the varying over the business cycle costs of financing, which together with their lower likelihood of deterioration in creditworthiness compared to low-rated borrowers points to their potentially lower through-the-cycle sensitivity to macroeconomic conditions.

Furthermore, McLean and Zhao (2014) show that in addition to direct implications, the business cycle related fluctuations in the availability and cost of financing have real, lasting effect on businesses of financially constrained low-rated firms, documenting that their investment and hiring are more sensitive to business cycle variations than those of high-rated firms. Moreover, using the survey data of Chief Financial Officers from the GFC, Campello et al. (2010) provide further evidence that the real costs of financial constraints are far greater for low-rated firms who respond by implementing deeper cuts to their technology, employment, and capital spending.^8^

This evidence suggests that financing constraints together with weakened business agility of low-rated firms at the start of an economic recovery can contribute to a lower level of resilience to macroeconomic shocks. Consequently, CDXHY is likely to be more sensitive than CDXIG to measures of macroeconomic conditions. Consistent with a lower resilience and a more challenging recovery prospect of lower-rated firms, we also posit that CDXHY is more sensitive to measures of macroeconomic uncertainty that can signal unstable recovery.

### Hypothesis 3

During an economic recovery, CDXHY is more sensitive than CDXIG to measures of economic output (H3a), employment conditions (H3b), inflation (H3c), and macroeconomic uncertainty (H3d).

Taken together, Hypotheses 1–3 provide arguments (a) for a close alignment of CDX spreads with measures of macroeconomic conditions, capturing both the economic state and associated uncertainty, and (b) against routinely employed conventional determinants fully capturing the macroeconomic influences relevant to CDX. Building on these arguments under informational efficiency, one should expect that CDX market participants would closely monitor macroeconomic conditions, promptly adjusting their CDX pricing when the conditions change. The CDX spread adjustments embedding such macroeconomic changes are expected to reflect both the direct influence channel (via the prices of risk) and the indirect channel (by anticipating future related changes in the firms’ financial and default-related policies). As such, CDX spreads should fully reflect macroeconomic conditions. Consequently, we posit that macroeconomic variables do not contain additional predictive information for future CDX spreads. Our analysis focuses on a quarter-ahead horizon as it is widely expected for a-quarter-ahead financial market prices to absorb changes in macroeconomic conditions. Our fourth hypothesis is formulated accordingly.

### Hypothesis 4

Macroeconomic variables do not contain predictive information for a-quarter-ahead CDX spreads.

## Analytical approach

We adopt the analytical framework of Ericsson et al. (2009) in that rather than conducting a full estimation of various structural models of interest, we examine a linear relationship between the theoretical determinants predicted by those models and CDX spreads.^9^ Our analysis is organized around our four hypotheses. We begin by introducing candidate macroeconomic variables, proceeding with a summary of conventional determinants.

### Macroeconomic variables

We employ a set of macroeconomic indicators together with measures of macroeconomic uncertainty, encompassing both output and employment aspects of real activity along with nominal activity, to capture the state of the economy. The selection of specific variables is guided by the recent theoretical models with macroeconomic influences and empirical evidence.

#### Economic indicators

*Industrial production growth* (IP) is among the most frequently utilized measures of output growth, employed, for example, in Baum and Wan (2010), Huang and Kong (2008), and Tang and Yan (2010). As IP is released at a monthly frequency, it is preferred over the GDP measure that is only available quarterly. Empirical evidence from these studies, aligned with the theoretical predictions in Tang and Yan (2006) and Chen (2010), suggests that IP negatively influences CDS spreads. As in the literature, we employ the year-on-year growth rate in industrial production.

*ISM Manufacturing Purchasing Managers Index (PMI)* is a survey-based forward-looking indicator signaling the purchasing managers’ outlook on the manufacturing sector of the economy. Gilbert et al. (2017) show that the information contained in PMI announcements is highly valuable for gauging the state of the economy in the U.S. relative to other macroeconomic indicators, with PMI announcements strongly impacting prices of U.S. Treasuries. Huang and Kong (2008) provide further evidence that PMI news announcements exert a pronounced influence on bond credit spreads. Consequently, we employ PMI in our analysis, being the first to assess its impact in the CDS market context. An increase in PMI, signaling an expansion of the manufacturing sector and a positive outlook for the overall economy, is expected to lower CDX spreads. In line with previous studies, we employ PMI measured in levels.

*Unemployment growth* (UG) is another real activity measure that captures labor market conditions; see, for example, Amato and Luisi (2006) and Zhou (2014). Interestingly, employing the noisy rational expectation model, Gilbert et al. (2017) find that the unemployment rate is among the few indicators that are most informative about the current state of the economy. Consequently, as investors are expected to put more weight on such indicator when forming their pricing expectations, we consider UG as our second novel macroeconomic indicator for CDS. Aligned with this prediction, Huang and Kong (2008) document that unemployment-related news announcements are particularly influential for corporate bond credit spreads. As an increase in UG signals worsening economic conditions, it is predicted to be positively related to the CDX spread. We employ the month-on-month percentage growth in the number of unemployed capturing the growth rate in unemployment.

*Total nonfarm payroll employment* (NFP), often referred to as nonfarm payroll for brevity, is a measure of labor market conditions that has been dubbed as “the king of announcements” in the financial press due to its releases known to exert a pronounced influence on various financial markets (Gilbert 2011). A potential explanation is that investors perceive nonfarm payroll announcements as a strong indicator of economic conditions, known to inform policy actions of the Federal Reserve (Gürkaynak and Wright 2013). Gilbert et al. (2017) and Huang and Kong (2008) provide empirical evidence of nonfarm payroll news impacting significantly Treasury yields and bond credit spreads, respectively. A rise in NFP, the third novel measure we employ in our CDS market context, indicating the workforce expansion by firms in expectation of strong economic growth, is predicted to lower the CDX spread. Consistent with the literature, we employ the month-on-month percentage change in nonfarm payroll.

*Consumer Price Index growth* (CPI) is employed as a measure of inflation. David (2008) predicts that high inflation widens credit spreads. Furthermore, Wu and Zhang (2008) among others provide empirical evidence of inflation being an important factor along with real activity for determining the term structure of bond credit spreads in the context of empirical no-arbitrage term structure models. In a contrasting set of results from unbalanced panel regressions, Tang and Yan (2010) do not find inflation important for firm-level CDS spreads. Given some ambiguity in the evidence to date, we proceed with assessing the impact of inflation for CDX index spreads. In line with the empirical literature, we employ the year-on-year percentage change in the consumer price index.

#### Economic uncertainty measures

The state of the economy is known to be important for determining the values of default-risk-sensitive securities such as CDS spreads. However, investors face uncertainty when assessing even the current economic state as indicators of real and nominal activity are released with delays. Consequently, such uncertainty ought to be reflected in CDX pricing.

We are the first to employ the Bloomberg ECOS data for measuring economic uncertainty. In contrast to previous studies, our nowcast-survey-based measures permit capturing various aspects of economic uncertainty. Furthermore, Andersen et al. (2003) and Swanson and Williams (2014) provide evidence that these data pass standard tests of forecast rationality, offering adequate measures of ex-ante expectations of the upcoming data release.

Moreover, our survey-based measures offer several advantages over both typically used financial-market-based measures such as the VIX index, known to be driven by time-varying investor risk aversion rather than economic uncertainty, and measures using economic historical time series data to construct uncertainty estimates. First, our uncertainty measures are model-free, hence, benefiting from flexibility in capturing uncertainty around economists’ subjective expectations about the state of the economy. The professional economists’ estimates are based on rich, most-up-to-date data and sophisticated analysis, utilizing a variety of econometric models and economic and financial data from a wide variety of sources (Zarnowitz and Braun 1993). In particular, Jo and Sekkel (2019) emphasize that such estimates naturally reflect potential time variation and structural changes in the economy, further pointing out that uncertainty associated with subjective professional economists’ estimates likely to matter more for investors’ decision making than alternative measures based on objective econometric model forecasts. Second, consensus survey-based estimates are formed in real time, using preliminary (unrevised) macroeconomic data announcements that also influence investors’ decision making. This contrasts with traditional econometric model forecasts using historical data that typically would have undergone several rounds of revision. Third, Ang et al. (2007) and Faust and Wright (2013) provide evidence that survey-based forecasts of macroeconomic fundamentals tend to be superior, particularly over the short-term horizons, to those based on econometric time-series models. This can be because uncertainty measures utilizing conditional volatility, like in Baum and Wan (2010), contain a foreseeable component that ought to be removed whereas GDP/IP growth forecast-error-based estimates as in Tang and Yan (2010) suffer from omitted-variable bias as their econometric-model forecasts use a pre-determined set of predictors limited to an AR(1) term only.

For each economic indicator, we construct two alternative uncertainty measures. More specifically, as in Dovern et al. (2012) and Popescu and Smets (2010), we employ the standard deviation (SD) and the interquartile range (IQR) of the cross-section of estimates supplied by professional economists for a forthcoming month release. The dispersion measures capture economists’ subjective uncertainty surrounding a current-month release, with the estimate submissions published by Bloomberg within a two-week window leading to the release day. Higher macroeconomic uncertainty signaled by these measures is expected to widen the spread.

Bloomberg ECOS economists supply their estimates for the current month’s IP growth, unemployment growth, and CPI growth, all measured as percentage growth relative to the previous month, the new nonfarm payroll employment, measured as the month-on-month change in the number of employed, and the level of PMI. Accordingly, we employ the SD (IQR) based measures of monthly cross-sections of these estimates as candidate determinants of CDX spreads, namely, *IPSD (IPIQR)*, *PMISD (PMIIQR)*, *URSD (URIQR)*, *NFPSD (NFPIQR)*, *CPISD (CPIIQR)*.^10^

### Conventional theoretical determinants

Ericsson et al. (2009) identify the determinants of credit spreads that are of central importance to the Merton (1974) approach, also commonly used in its many extensions. We employ similar conventional determinants, adapting their firm-specific measures to our index-portfolio context.

Accordingly, our first variable measures the firm’s asset value growth, a central determinant for default-risk-sensitive securities. This is because in the structural models the firm defaults when the firm’s asset value falls below a certain threshold. Consequently, a higher drift in the firm’s asset value process lowers the likelihood of default by pulling the firm’s asset value away from the default threshold. As the firm’s asset value is not directly observable, we follow the voluminous literature employing *the stock market equity return (ER)*, assuming it approximates reasonably well the firm’s asset value growth. For example, Tang and Yan (2010) and Shi et al. (2022) document a significant negative influence of the stock market return on the cross-section average of firm-level CDS spreads and synthetic CDS index spread changes, respectively. Similarly, Blanco et al. (2005), Baum and Wan (2010), and Pires et al. (2015) find a negative relationship between the single-name CDS spread and the return on the firm’s equity for both the U.S and European CDS. In line with these studies, we employ the monthly S&P 500 market return. Monthly index levels, entering the return calculations, are obtained by averaging over daily observations within a given month.

Our second variable, *equity volatility (EV)* is of importance in structural models as higher equity volatility, implying higher volatility of the firm’s asset value, increases the likelihood of the asset value crossing the default threshold; see, for example, Alexander and Kaeck (2008). This is because the default-risky security is equivalent to the default-risk-free security combined with a short put on the firm’s asset value, with volatility influencing the value of the put option. Consequently, higher equity volatility is associated with a higher CDS spread. Annaert et al. (2013), Baum and Wan (2010), Galil et al. (2014), Sun et al. (2021) inter alia provide evidence of the positive effect of volatility in equity returns for both U.S. and European CDS. Irresberger et al. (2018) further document that market-wide implied equity volatility remains important for determining CDS spreads even after accounting for firm-specific equity volatility. Accordingly, we employ VIX, the option-implied volatility index of the S&P 500, as our equity volatility measure, with monthly observations obtained by averaging over daily values in a month.

*The risk-free interest rate (RF)* is the third essential determinant of the CDS spread. In the Merton (1974) model, a higher risk-free interest rate increases the drift of the risk-neutral firm value process, thus, lowering the likelihood of default. Accordingly, the risk-free rate is predicted to negatively influence CDX spreads, with voluminous empirical literature evidencing the importance of the risk-free rate for CDS spreads. As in Collin-Dufresne et al. (2001), Blanco et al. (2005), and Ericsson et al. (2009) inter alia, we employ the 10-year U.S. Treasury rate as our measure of the risk-free interest rate. We obtain monthly values by averaging over daily values within a month.

## Data

The Bloomberg ECOS data, employed for constructing our measures of economic uncertainty, contains nowcasts for key economic indicators made by professional economists up to one day before each major data release. Table 1 shows that each indicator attracts, on average, between 77 and 87 monthly nowcast submissions per indicator. Even though the number of submissions experiences some variation month-on-month, it remains sufficiently high each month. Nonfarm payroll attracts the highest number of monthly submissions, ranging between 70 and 100, with the unemployment rate submissions, ranging between 68 and 92, appearing the second highest, and the industrial production submissions, ranging between 60 and 87, among the lowest.

**Table 1:** Number of professional economists nowcasts

Indicator	Mean	Median	Min	Max
IP	78	79	60	87
PMI	77	78	63	88
UR	82	83	68	92
NFP	87	87	70	100
CPI	80	80	62	89

The table gives the mean, median, minimum and maximum number of economists supplying their estimates to ECOS, Bloomberg for industrial production growth (IP), ISM Manufacturing Purchasing Managers Index (PMI), unemployment rate (UR), total nonfarm payroll employment (NFP) and growth in the consumer price index (CPI). The time period is from July 2009 to December 2018

Our CDXIG and CDXHY data come from Bloomberg.^11^ CDXIG and CDXHY comprise 125 and 100 equally-weighted investment-grade and high-yield names, respectively. Each index represents a basket of the most liquid single-name CDS contracts of 5-year maturity in a relevant credit grade category. For example, on average, 226 and 276 daily transactions were recorded for CDXIG and CDXHY, respectively, in 2018 (Boyarchenko et al. 2020). We collect daily end-of-day mid-spreads, the average between the bid and ask quotes, stamped with New York time. As in Tang and Yan (2010) and Baum and Wan (2020) among others, monthly observations are obtained by averaging daily CDX spread quotes within a month.

**Fig. 1:**
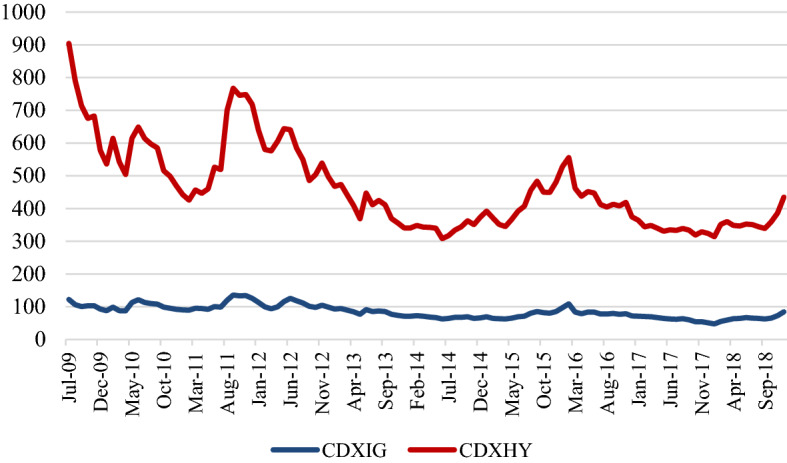
Evolution of CDXIG and CDXHY spreads.CDXIG and CDXHY spreads both are given in basis points. The time period is from July 2009 to December 2018

Figure 1 presents the evolution of the CDXIG and CDXHY spreads over our sample period, which coincides with the period of economic recovery following the GFC. The figure highlights a substantial co-movement of the two indices, exhibiting very similar trends over time. Nevertheless, the differences exist, reflecting CDX credit quality. This is further highlighted in the summary of descriptive statistics in Table 2. The table shows that the average spread of 460.20 basis points (bps) for speculative-grade CDXHY is over fivefold higher than the investment-grade CDXIG spread, averaging at 84.33 bps. The differences in volatility are even more pronounced, with the standard deviation and the range for CDXHY, both over sixfold higher than those for CDXIG. In addition, a relatively high autocorrelation coefficient of around 0.9 evidences the stylized persistency in both CDX spreads. Nevertheless, the Augmented Dickey-Fuller test results indicate stationarity. Table 3 further reports pairwise correlation coefficients for our data variables.

**Table 2:** Summary statistics of CDX spreads and conventional and macroeconomic determinants

Variable	Mean	Median	Maximum	Minimum	Std. Dev	AR(1)	ADF test statistic
*CDX spreads*							
CDXIG	84.33	83.11	135.62	46.90	20.16	0.92	− 3.32*
CDXHY	460.20	430.13	904.22	307.63	127.85	0.90	− 2.93**
*Conventional determinants*							
RF	2.49	2.38	3.85	1.50	0.59	0.94	− 2.60*
ER	0.94	1.37	7.90	− 10.56	2.79	0.14	− 8.82***
EV	17.41	16.19	36.53	10.13	5.50	0.79	− 3.52***
*Macroeconomic indicators*							
IP	2.35	3.20	8.20	− 13.10	3.21	0.85	− 5.88***
PMI	54.73	54.75	61.40	48.20	3.53	0.82	− 3.41**
UG	− 0.71	− 0.60	8.23	− 6.99	2.48	− 0.15	− 9.21***
NFP	0.10	0.12	0.33	− 0.20	0.09	0.56	− 5.08***
CPI	1.67	1.70	3.90	− 2.10	1.06	0.88	− 3.95***
*Macroeconomic uncertainty*							
IPSD	0.20	0.20	0.55	0.11	0.06	0.29	− 9.45***
IPIQR	0.26	0.21	0.70	0.10	0.09	0.31	− 8.64***
PMISD	0.75	0.72	1.25	0.45	0.17	0.52	− 5.83***
PMIIQR	0.95	1.00	1.80	0.40	0.28	0.38	− 7.00***
URSD	0.06	0.06	0.11	0.03	0.02	0.58	− 5.57***
URIQR	0.06	0.10	0.20	0.00	0.05	0.08	− 9.76***
NFPSD	0.02	0.02	0.08	0.01	0.01	0.58	− 5.48***
NFPIQR	0.02	0.02	0.09	0.01	0.01	0.52	− 6.01***
CPISD	0.09	0.08	0.15	0.03	0.02	0.47	− 6.47***
CPIIQR	0.09	0.10	0.20	0.00	0.05	0.16	− 9.15***

The table gives descriptive statistics of CDXIG and CDXHY index spreads, conventional determinants: 10-year risk-free interest rate (RF), equity return (ER), measured by the return on the S&P500 index, and equity volatility (EV), measured by the CBOE equity volatility VIX index, and macroeconomic series: industrial production growth (IP), ISM Manufacturing Purchasing Managers Index (PMI), unemployment growth (UG), total nonfarm payroll employment (NFP) and growth in the consumer price index (CPI) together with the standard deviation (SD) and inter-quartile-range (IQR) based uncertainty measures associated with each of the fundamentals. Daily data for CDXIG and CDXHY comes from Bloomberg. Daily data for RF and ER comes from Federal Reserve Economic Data and from Chicago Board Options Exchange for VIX. Daily series are converted into monthly by averaging over daily observations in a given month. All macroeconomic data is available at monthly frequency. Data for IP and PMI are collected from the Board of Governors of the Federal Reserve System and the Institute for Supply Management, respectively. Data for UG, NFP and CPI come from the U.S. Bureau of Labor Statistics. Monthly data for all macroeconomic uncertainty measures comes from Bloomberg. The time period spans from July 2009 to December 2018

**Table 3:** Variable pairwise correlation

Variables	CDXIG	CDXHY	RF	ER	EV	IP	PMI	UG	NFP	CPI	IPSD	IPIQR	PMISD	PMIIQR	URSD	URIQR	NFPSD	NFPIQR	CPISD	CPIIQR
CDXIG	1.00																			
CDXHY	0.92	1.00																		
RF	− 0.01	0.12	1.00																	
ER	− 0.12	− 0.07	0.04	1.00																
EV	0.80	0.87	0.26	0.00	1.00															
IP	− 0.07	− 0.30	0.02	− 0.16	− 0.12	1.00														
PMI	− 0.41	− 0.37	0.59	0.10	− 0.18	0.44	1.00													
UG	0.08	0.11	0.01	0.02	0.12	− 0.11	− 0.06	1.00												
NFP	− 0.42	− 0.56	− 0.33	− 0.03	− 0.43	0.41	0.11	0.00	1.00											
CPI	0.08	− 0.06	0.09	− 0.12	0.00	0.52	0.35	− 0.09	0.23	1.00										
IPSD	0.16	0.29	0.12	− 0.03	0.13	− 0.39	− 0.10	− 0.13	− 0.37	− 0.30	1.00									
IPIQR	0.22	0.39	0.14	− 0.05	0.26	− 0.41	− 0.11	− 0.14	− 0.36	− 0.29	0.82	1.00								
PMISD	0.20	0.20	0.42	− 0.07	0.27	0.12	0.21	0.13	− 0.26	0.12	0.13	0.11	1.00							
PMIIQR	0.21	0.15	0.37	− 0.03	0.20	0.14	0.21	0.12	− 0.12	0.09	0.00	− 0.03	0.69	1.00						
URSD	0.29	0.33	0.46	0.14	0.32	− 0.05	0.09	0.01	− 0.30	− 0.08	0.21	0.26	0.26	0.30	1.00					
URIQR	0.07	0.09	0.09	0.13	0.08	− 0.04	0.02	0.00	0.02	0.02	0.17	0.22	0.15	0.14	0.53	1.00				
NFPSD	0.38	0.44	0.49	− 0.04	0.45	0.02	0.17	− 0.03	− 0.22	− 0.03	0.37	0.36	0.42	0.32	0.51	0.22	1.00			
NFPIQR	0.40	0.47	0.49	− 0.10	0.47	0.00	0.15	− 0.02	− 0.30	0.04	0.38	0.39	0.44	0.30	0.50	0.25	0.95	1.00		
CPISD	0.36	0.36	− 0.07	− 0.03	0.27	− 0.14	− 0.35	− 0.04	− 0.21	− 0.18	0.36	0.30	0.18	0.14	0.11	0.03	0.24	0.24	1.00	
CPIIQR	0.31	0.34	0.02	− 0.03	0.26	− 0.09	− 0.18	0.00	− 0.20	− 0.14	0.34	0.28	0.17	0.13	0.12	0.07	0.21	0.26	0.66	1.00

**Fig. 2:**
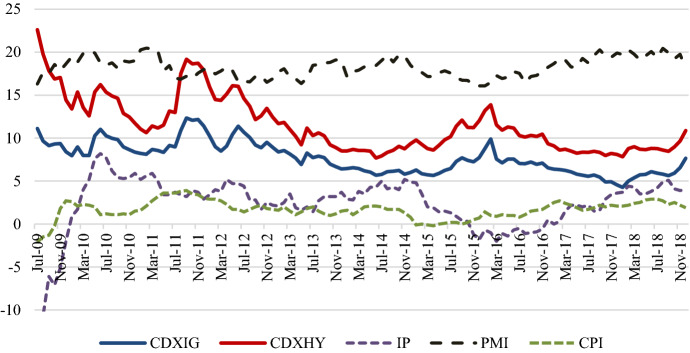
Evolution of CDX spreads and selected macroeconomic indicators. The figure gives time series of CDXIG and CDXHY spreads together with IP, CPI and PMI macroeconomic series. To enable the comparison, CDXIG, CDXHY and PMI series have been scaled by 1/40, 1/11, and 1/3 respectively. The time period is from July 2009 to December 2018

Figure 2 contrasts the dynamics of CDX spreads and selected macroeconomic fundamentals. The figure shows that IP and PMI both exhibit countercyclical dynamics to that of CDX spreads, with CPI initially showing a positive co-movement but then becoming rather decoupled from CDX over time.^12^ Table 2 further provides descriptive statistics for the macroeconomic variables. IP, PMI, and CPI series, all show high persistence, which is notably higher than that of labor market variables, NFP and UG, as expected. At the same time, NFP-related uncertainty exhibits the strongest persistence among the uncertainty measures.

Table 2 further reveals that the mean and the median values of SD and IQR measures of uncertainty are consistent across various indicators in that an indicator with a relatively high mean (median) value for SD tends to also have a high IQR value. For example, NFPSD and NFPIQR have the lowest mean, both at 0.02, across the uncertainty measures whereas PMISD and PMIIQR are characterized by the highest mean at 0.75 and 0.95, respectively. As expected, IQR-based measures tend to have higher mean and median values relative to SD-based measures. However, IQR measures are also characterized by a notably higher variability as evidenced by both relatively high standard deviation and wide range of values that IQR measures take compared to SD measures, even after adjusting for size.

## Regression analysis

### Regression methodology

Our regression analysis focuses on testing *Hypothesis **1*, *Hypothesis **2*, and *Hypothesis **3*.

Informed by *Hypothesis **1*, the first regression model links ${CDX}_{i,t}$, $i=\left\{CDXIG, CDXHY\right\}$ to the vector of macroeconomic indicators, ${M}_{t}\equiv \left({IP}_{t}, {PMI}_{t}, {UG}_{t}, {NFP}_{t}, {CPI}_{t}\right)$, and the vector of macroeconomic uncertainty measures, ${U}_{j,t}$, $j=\mathrm{1,2},$ resulting in the following regression model:

1-2 $$CDX_{i,t} = \alpha_{i} + \beta_{i}^{M} M_{t} + \beta_{i,j}^{U} U_{j,t} + \varepsilon_{i,t}$$

We entertain two alternative specifications of the vector of macroeconomic uncertainty measures, namely, $U_{1,t} \equiv \left( {IPSD_{t} , PMISD_{t} , URSD_{t} , NFPSD_{t} , CPISD_{t} } \right)$ that enters Eq. (1) and $U_{2,t} \equiv \left( {IPIQR_{t} , PMIIQR_{t} , URIQR_{t} , NFPIQR_{t} , CPIIQR_{t} } \right)$ utilized in Eq. (2).

In testing *Hypothesis **2*, we begin with the baseline regression model that links CDX spreads to a vector of conventional theoretical determinants $F_{t} \equiv \left( {RF_{t} , ER_{t} , EV_{t} } \right)$ only^13^:

3 $$CDX_{i,t} = \alpha_{i} + \beta_{i}^{F} F_{t} + \varepsilon_{i,t}$$

At the next stage, we augment the baseline model successively with the vector of macroeconomic indicators and the vector of uncertainty measures, resulting in the following model specification:

4-5 $$CDX_{i,t} = \alpha_{i} + \beta_{i}^{F} F_{t} + \beta_{i}^{M} M_{t} + \beta_{i,j}^{U} U_{j,t} + \varepsilon_{i,t}$$

Here Eq. (4) utilizes the vector of SD-based uncertainty measures, ${U}_{1,t}$, and the vector of IQR-based measures, ${U}_{2,t}$, enters Eq. (5).

Using the monthly series of CDX index spreads and macroeconomic variables, the coefficients in regression Eqs. (1) – (5) are estimated by OLS, employing the t-statistics with Newey-West standard errors, robust to the presence of serial correlation and heteroskedasticity.^14^

In testing *Hypothesis **3*, we employ the model linking CDX spreads to macroeconomic variables, Eqs. (1) and (2), along with the model employing the conventional determinants together with macroeconomic variables, Eqs. (4) and (5). This is to limit potential bias related to the conventional determinants capturing at least some macroeconomic influences in our CDX index context. Moreover, as the mean CDXHY spread is over five-fold greater than the mean CDXIG spread (Table 2), the regression coefficients in Eqs. (1, 2, 4, and 5) require adjusting for testing this hypothesis. Consequently, the sensitivity coefficients are calculated by dividing the regression coefficient of an explanatory variable by the mean of the dependent variable and multiplying by the mean of the explanatory variable.

### Regression analysis

#### CDX spreads and the macroeconomy

Table 4 presents the results of regressions (1) and (2). The results in Panel A indicate that all five macroeconomic variables significantly influence CDXIG. PMI and NFP exert the most pronounced impact: a typical change, approximated by a one-standard-deviation increase in the variable at hand, narrows the spread by 12 bps and 8 bps, respectively, both at the 1% significance level. A similar change in CPI, IP, and UG widens the spread by about 6 bps, 4 bps, and 2 bps, respectively, at the 5–10% significance level. Contrary to the prediction in the previous literature for CDS of any credit quality, we document a positive impact of IP on CDXIG. This result is aligned with the empirical findings for bond credit spreads of high (but not low) credit quality in Amato and Luisi (2006) and Wu and Zhang (2008). As along with IP, capturing historical output growth, our analysis employs forward-looking PMI that exerts a strong negative impact on CDXIG as expected, the regression coefficient of IP can be reflective of the remaining, potentially historical dependency of CDXIG on the economic output growth.^15^

**Table 4:** Macroeconomic determinants of CDXIG and CDXHY spreads

	Panel A: CDXIG spread					Panel B: CDXHY spread			
Variable	Equation (1)		Equation (2)			Equation (1)		Equation (2)	
	Coeff	t-Stat	Coeff	t-Stat		Coeff	t-Stat	Coeff	t-Stat
*Macroeconomic indicators*									
IP	1.21	2.01**	1.37	2.14**		− 2.04	− 0.53	− 0.14	− 0.03
PMI	− 3.37	− 4.86***	− 3.48	− 4.71***		− 14.91	− 3.58***	− 15.41	− 3.55***
UG	0.82	2.10**	0.72	2.03**		6.30	2.61**	6.39	3.16***
NFP	− 92.11	− 5.60***	− 80.04	− 4.56***		− 656.07	− 6.52***	− 560.11	− 4.97***
CPI	6.00	2.45**	5.38	2.02**		30.89	1.80*	27.27	1.52
*Macroeconomic uncertainty*									
IPSD	− 15.54	− 0.68				− 70.43	− 0.43		
IPIQR			16.93		0.94			193.16	1.53
PMISD	− 4.24	− 0.48				− 50.01	− 0.85		
PMIIQR			9.66		1.64			24.68	0.82
URSD	92.72	0.79				413.08	0.52		
URIQR			− 6.61		− 0.28			− 70.26	− 0.50
NFPSD	870.32	5.42***				6110.36	6.34***		
NFPIQR			514.97		3.72***			3578.52	4.74***
CPISD	71.13	1.31				488.73	1.39		
CPIIQR			27.70		1.16			208.12	1.65
Intercept	243.87	6.38***	243.26		6.16***	1168.50	5.00***	1146.53	4.93***

	Panel C: CDXIG spread			Panel D: CDXHY spread				
Variable blocks	R^2^	R^2^ adj	Partial R^2^	R^2^	R^2^ adj	Partial R^2^		
Macroeconomic indicators: 5-vector; eqs. (1, 2)	0.453	0.427		0.454	0.428			
Macroeconomic uncertainty: 5-vector of SD measures; Eq. (1)	0.607	0.568	0.281***	0.627	0.591	0.317***		
Macroeconomic uncertainty: 5-vector of IQR measures; Eq. (2)	0.596	0.557	0.262***	0.617	0.580	0.300***		

The table presents the regression estimation results for eqs. (1) and (2) for the CDXIG spread in Panel A and the CDXHY spread in Panel B. Regression Eqs. (1) and (2) in both panels employ SD and IQR based measures of uncertainty, respectively. Panels C and D provide R^2^, adjusted R^2^, and partial R^2^ from regressing the CDXIG spread (Panel C) and the CDXHY spread (Panel D) on variable blocks $.$ The first row reports R^2^ and adjusted R^2^ for the regression employing the block of macroeconomic indicators, *M*_*t*_, alone. The second and third rows report R^2^ and adjusted R^2^ for the regression augmented with the SD-based measures of uncertainty, *U*_*1,t*_, (Eq. 1) and the IQR-based measures of uncertainty, *U*_*2,t*_, (Eq. 2), respectively, followed by the partial R^2^ for the block of uncertainty measures at hand. The significance of the partial R^2^ values is based on the *p-*values of the partial *F*-statistic of the block of variables. *, **, ***denote significance at 10%, 5% and 1% levels, respectively. The sample period is from July 2009 to December 2018

**Table 5:** Conventional and macroeconomic determinants of CDXIG and CDXHY spreads

	Panel A: CDXIG spread						Panel B: CDXHY spread					
Variable	Eq. (3)		Eq. (4)		Eq. (5)		Eq. (3)		Eq. (4)		Eq. (5)	
	Coeff.	t-Stat.	Coeff.	t-Stat.	Coeff.	t-Stat.	Coeff.	t-Stat.	Coeff.	t-Stat.	Coeff.	t-Stat.
*Conventional determinants*												
RF	− 7.63	− 1.62	− 6.01	− 1.97*	− 5.28	− 1.75*	− 23.86	− 1.18	− 32.64	− 1.78*	− 24.91	− 1.38
ER	− 0.78	− 2.12**	− 0.33	− 1.16	− 0.17	− 0.55	− 3.11	− 1.25	− 3.13	− 2.00**	− 2.02	− 1.18
EV	3.42	10.83***	2.44	9.11***	2.45	10.20***	22.64	9.30***	17.21	12.01***	17.08	12.42***
*Macroeconomic indicators*												
IP			0.75	1.65	0.74	1.64			− 5.03	− 2.09**	− 4.24	− 1.63
PMI			− 1.75	− 2.70***	− 1.91	− 3.11***			− 4.31	− 1.52	− 5.55	− 1.96*
UG			0.22	0.70	0.02	0.08			2.02	1.19	1.53	0.95
NFP			− 46.52	− 2.90***	− 43.87	− 3.22***			− 318.89	− 3.66***	− 290.95	− 3.69***
CPI			4.18	2.89***	3.71	2.45**			17.76	3.10***	15.51	2.33**
*Macroeconomic uncertainty*												
IPSD			9.18	0.54					111.19	1.11		
IPIQR					5.47	0.51					114.78	1.61
PMISD			− 1.56	− 0.22					− 36.30	− 0.99		
PMIIQR					8.48	2.02**					12.34	0.67
URSD			118.72	1.68*					553.33	1.20		
URIQR					1.76	0.10					− 2.04	− 0.02
NFPSD			321.40	2.52**					2041.74	3.85***		
NFPIQR					202.83	2.14**					1188.30	3.16***
CPISD			33.72	0.68					227.90	0.88		
CPIIQR					13.51	0.67					113.71	1.34
Intercept	104.06	7.82***	174.66	5.30***	183.15	5.98***	522.55	9.26***	709.04	5.24***	763.29	5.69***

	Panel C: CDXIG spread			Panel D: CDXHY spread								
Variable blocks	R^2^	R^2^ adj.	Partial R^2^	R^2^	R^2^ adj.	Partial R^2^						
Conventional determinants: 3-vector; eqs. (3-5)	0.698	0.690		0.765	0.758							
Macroeconomic indicators: 5-vector; eqs. (4, 5)	0.781	0.765	0.275***	0.851	0.840	0.366***						
Macroeconomic uncertainty: 5-vector of SD measures; eq. (4)	0.810	0.786	0.132**	0.879	0.863	0.189***						
Macroeconomic uncertainty: 5-vector of IQR measures; eq. (5)	0.808	0.783	0.123**	0.873	0.856	0.147***						

The table presents the regression estimation results of eqs. (3) – (5) for the CDXIG spread in Panel A and the CDXHY spread in Panel B. Regression Eqs. (4) and (5) in both panels employ SD and IQR based measures of uncertainty, respectively. Panels C and D provide R^2^, adjusted R^2^, and partial R^2^ from regressing the CDXIG spread (Panel C) and the CDXHY spread (Panel D) on variable blocks. The first row reports R^2^ and adjusted R^2^ for the regression employing the block of conventional determinants, *F*_*t*_*,* alone (Eq. 3). The subsequent rows report R^2^ and adjusted R^2^ for the regression successively augmented with the block of macroeconomic indicators, *M*_*t*_, (row two) and the block of macroeconomic uncertainty measures: *U*_*1,t*_, Eq. (4) (row three) or *U*_*2,t*_. Equation (5) (row four), each followed by the partial R^2^ for the variable block at hand. The significance of the partial R^2^ values is based on the *p-*values of the partial *F*-statistic of the block of variables. *, **, *** denote significance at 10%, 5% and 1% levels, respectively. The sample period is from July 2009 to December 2018

At the same time, four out of five variables: PMI, UG, NFP, and CPI significantly affect CDXHY in Panel B. Similar to CDXIG, PMI and NFP exert the strongest impact on CDXHY: a one-standard-deviation increase in these variables narrows the spread by about 53 bps and 58 bps, respectively, both at the 1% significance level. A similar increase in CPI and UG widens the spread by about 33 bps and 17 bps, at the 5–10% significance level. Interestingly, IP, commonly used to capture the state of the economy, has the expected negative sign but is not significant for CDXHY which suggests that other variables might be better candidates. Panels C and D further show that macroeconomic indicators jointly explain a sizable, about 43%, share of variation in spreads for both CDXIG and CDXHY.

Turning to macroeconomic uncertainty measures, the regression results for Eqs. (1) and (2) unambiguously indicate the importance of NFPSD and NFPIQR, respectively, in determining CDX spreads, with both measures significant at the 1% level for CDXIG and CDXHY. The economic impact of NFPSD, which is slightly more pronounced than that of NFPIQR, is sizable and comparable to that of NFP for both CDXIG and CDXHY, with a one-standard-deviation increase in NFPSD widening CDXIG and CDXHY spreads by about 8 bps and 53 bps, respectively. The additional joint contribution of uncertainty measures to the R^2^ value is also notable, with these measures able to explain over 26% and 30% of the residual variation, not explained by the macroeconomic indicators, for CDXIG and CDXHY, respectively. These findings altogether provide evidence in support of *Hypothesis **1*.

#### Macroeconomic influences and conventional determinants

Next, we turn to testing *Hypothesis **2*. Regression results for Eqs. (4) and (5) in Table 5 provide evidence that even though the conventional determinants in Eq. (3) can explain a large share of spread variation for both CDXIG and CDXHY, nevertheless, four macroeconomic variables: PMI, NFP, CPI, and NFP-related uncertainty retain their significance for CDXIG in both Eqs. (4) and (5) in Panel A, after accounting for the impact of the conventional determinants. For CDXHY in Panel B, the results are even more striking as most macroeconomic variables, identified as important in our previous analysis, namely, PMI, NFP, and NFP-related uncertainty measures (but not UG) remain significant, albeit with the significance level somewhat lower for PMI.

The results in Panels C and D provide further evidence for the economic relevance of macroeconomic variables that significantly contribute to the R^2^ value for both CDX spreads. Specifically, macroeconomic indicators jointly explain 27.5% and 36.6% of the residual variation, not attributed by the conventional variables, for CDXIG and CDXHY, respectively. The additional contribution to the R^2^ value of the uncertainty measures drops roughly by half when the conventional determinants are added to the regression equation, yet like for the macroeconomic set of indicators, it remains statistically significant and notable in size, for instance, at 13.2% for CDXIG and 18.9% for CDXHY in Eq. (4), employing SD-based measures of uncertainty.

All-in-all, our results unequivocally indicate that macroeconomic indicators and uncertainty measures both significantly influence CDXIG and CDXHY spreads, beyond the impact of the conventional determinants, providing evidence in support of *Hypothesis **2*.

As the influence of SD-based measures of uncertainty is notably more pronounced relative to IQR-based measures for both CDX indices, leading to a consistently higher contribution to the R^2^ value across the analyses in Tables 4 and 5, we focus on the SD measures of uncertainty in our forthcoming analysis in Sect. 6.

#### Sensitivity to macroeconomic influences

A higher contribution of uncertainty measures to the explained variation in CDXHY spreads relative to CDXIG spreads (Tables 4 and 5) can be related to a higher sensitivity of lower credit-quality CDX to economic uncertainty. As sensitivity to other variables can also differ for CDXIG and CDXHY (*Hypothesis **3*), Table 6 provides the results of the sensitivity analysis. The results for Eqs. (1) and (2) unambiguously indicate that CDXIG is more sensitive than CDXHY to measures of economic output, contrary to *H3a*. Specifically, IP impacts significantly CDXIG but not CDXHY, with CDXIG also revealing 1.2-fold greater sensitivity to PMI. Furthermore, CDXIG displays a marginally higher sensitivity than CDXHY to CPI, contrary to *H3c*. In contrast, CDXHY is 1.3 to 1.6-fold more sensitive than CDXIG to employment indicators, NFP and UG, providing evidence for *H3b*. The sensitivity to both NFPSD and NFPIQR is also approximately 1.3 times greater for CDXHY than CDXIG, in line with *H3d*. These findings are generally consistent with the analysis based on Eqs. (4) and (5), augmented with conventional regressors, also reported in Table 6.^16^

**Table 6:** Sensitivity of CDXIG and CDXHY spreads to macroeconomic variables

	Equation (1)		Equation (2)		Equation (4)		Equation (5)	
Variable	CDXIG spread	CDXHY spread	CDXIG spread	CDXHY spread	CDXIG spread	CDXHY spread	CDXIG spread	CDXHY spread
*Conventional determinants*								
RF					− **0.177**	− **0.177**	− **0.156**	− 0.135
ER					− 0.004	− **0.006**	− 0.002	− 0.004
EV					**2.125E-17**	**2.752E-17**	**2.142E-17**	**2.732E-17**
*Macroeconomic indicators*								
IP	**0.034**	− 0.010	**0.038**	− 0.001	0.021	− **0.026**	0.021	− 0.022
PMI	− **2.187**	− **1.773**	− **2.258**	− **1.832**	− **1.136**	− 0.512	− **1.240**	− **0.660**
UG	− **0.007**	− **0.010**	− **0.006**	− **0.010**	− 0.002	− 0.003	0.000	− 0.002
NFP	− **0.115**	− **0.149**	− **0.099**	− **0.128**	− **0.058**	− **0.073**	− **0.055**	− **0.066**
CPI	**0.119**	**0.112**	**0.107**	0.099	**0.083**	**0.065**	**0.074**	**0.056**
*Macroeconomic uncertainty*								
IPSD	− 0.038	− 0.031			0.022	0.049		
IPIQR			0.052	0.108			0.017	0.064
PMISD	− 0.038	− 0.082			− 0.014	− 0.059		
PMIIQR			0.109	0.051			**0.096**	0.026
URSD	0.066	0.054			**0.085**	0.072		
URIQR			− 0.005	− 0.009			0.001	0.000
NFPSD	**0.202**	**0.259**			**0.074**	**0.087**		
NFPIQR			**0.149**	**0.190**			**0.059**	**0.063**
CPISD	0.072	0.091			0.034	0.042		
CPIIQR			0.029	0.040			0.014	0.022

The table gives the sensitivity coefficients for the macroeconomic indicators and measures of macroeconomic uncertainty corresponding to the regression estimation results reported in Tables 4 and 5. The first column lists the explanatory variables. Columns two and three give the sensitivity coefficients corresponding to Eq. (1) for CDXIG and CDXHY, respectively. Columns four and five give the respective sensitivity coefficients related to Eq. (2) for the CDXIG spread and the CDXHY spread. Similarly, columns 6, 7, 8, and 9 report the respective sensitivity coefficients for CDXIG and CDXHY, corresponding to Eqs. (4) and (5). For a given regression equation, the sensitivity coefficients are calculated by dividing the slope coefficient of an explanatory variable at hand by the mean of the dependent variable and multiplying by the mean of the explanatory variable. Coefficients that are significant at least at the 10% level in the corresponding regressions are highlighted in bold

A possible explanation for a higher sensitivity of CDXIG to economic output and its marginally higher sensitivity to inflation is that during an economic recovery business outlook of high-rated firms can be closer aligned with longer-term economic trends, signaled by more persistent economic output and inflation measures. This is due to the ability of high-rated firms, benefitting from continued access to favorable financing terms, to utilize their relatively intact assets, workforce, and cash reserves to take on profitable investment opportunities post-crisis. In contrast, weakened business agility of low-rated firms at the start of an economic recovery, caused by the depletion of assets, workforce, and internal cash reserves, can hinder their ability to take on profitable investment opportunities (e.g., Campello et al. 2010; McLean and Zhao 2014). Moreover, these factors, exacerbated by financing constraints, would also contribute to their lower level of resilience to even short-lived macroeconomic shocks or slower than expected economic recovery. This can explain high CDXHY sensitivity relative to CDXIG to more flexible macroeconomic measures such as employment indicators that can signal delayed or faltering recovery. A lower resilience and a more challenging recovery prospect of lower-rated firms would also make them more sensitive to measures of macroeconomic uncertainty.

Furthermore, only a marginal difference in the sensitivity of CDXIG and CDXHY to inflation can be linked to a limited informational content of the inflation indicator in our post-GFC data sample that covers a period of low and stable inflation. This is supported by a weaker impact of CPI on both CDXIG and CDXHY relative to PMI and NFP and consistent with the analysis in Amato and Luisi (2006) who document a relatively low impact of inflation on bond credit spreads during an earlier period of low and stable inflation that they study.

Moreover, our findings help explain some conflicting results reported for similar regimes of relative economic stability in Kim et al. (2017) and Chan and Marsden (2014) that can be linked to the authors’ choice of business cycle measures, representing a combination of economic and market variables. Specifically, a key measure in Kim et al. (2017), the expected market risk premium, is constructed using data predominantly reflecting investment-grade entities such as the default premium on investment-grade (Baa-rated) bonds and the aggregate dividend yield on the CRSP value-weighted portfolio together with the government bond data. Such risk premium measure, by construction, reflects risk assessments largely associated with investment-grade borrowers that, as our results suggest, differ from speculative-grade borrowers in their sensitivity to fundamentals and exhibit notably lower sensitivity to macroeconomic uncertainty. Thus, employing such measures is likely to lead to an underestimation of macroeconomic influences on speculative-grade firms reflected in the high-yield CDS spreads, explaining their lower impact on high-yield CDS relative to investment-grade CDS in Kim et al. (2017). At the same time, utilizing generic macroeconomic indices, based on data aggregation over a broad range of fundamentals, such as the ADS index in Chan and Marsden (2014), can lead to averaging out of some of the macroeconomic influences for both CDXIG and CDXHY, potentially leading to the underestimation of the effect on both CDX indices. Moreover, the use in both studies of additional market variables, all influenced by the underlying macroeconomy, can introduce complex multicollinearity issues as recognized in Kim et al. (2017).^17^

Furthermore, as a robustness check, we have repeated the analysis in Sect. 5, as reported in Tables 4–6, using the end-of-month data for CDX spreads and other financial market variables. The results, available upon request, are generally consistent with those reported here.

## Forecasting analysis

### Forecasting methodology

As the variable significance in-sample does not directly translate into the predictive ability OOS, or in real time, our analysis proceeds with evaluating the predictive content of economic variables for future CDX spreads by means of OOS forecasting, using a hierarchical regression approach. Our OOS forecasting exercise consists of two parts.

The first part focuses on testing *Hypothesis **4* by assessing whether macroeconomic variables and uncertainty measures have predictive ability over and beyond that of the conventional theoretical predictors. Consequently, for our *Baseline 1* model, aligned with *Hypothesis **4*, we employ a predictive model based on Eq. (3) that uses only the conventional variables as predictors, constructing the h-steps ahead forecasts using the following equation:

6 $$CDX_{i,t + h} = \alpha_{i} + \gamma_{i}^{F} F_{t} + \varepsilon_{i,t + h}$$

In our hierarchical approach, we first successively add, one-by-one, macroeconomic indicators, leading to the predictive model:

7 $$CDX_{i,t + h} = \alpha_{i} + \gamma_{i}^{F} F_{t} + {\varvec{\gamma}}_{{\varvec{i}}}^{{\varvec{M}}} {\varvec{M}}_{{\varvec{t}}} + \varepsilon_{i,t + h}$$

before further gradually augmenting the predictive model by measures of economic uncertainty:

8 $$CDX_{i,t + h} = \alpha_{i} + \gamma_{i}^{F} F_{t} + \gamma_{i}^{M} M_{t} + {\varvec{\gamma}}_{{\varvec{i}}}^{{\varvec{U}}} {\varvec{U}}_{{\varvec{t}}} + \varepsilon_{i,t + h}$$

Given the stylized persistence of CDS spreads, in the second part we modify our baseline model specification by adding an AR(1) term. The resulting *Baseline 2* model permits assessing the predictive ability of macroeconomic and uncertainty variables beyond the information already reflected in today’s CDX spread along with conventional predictors^18^:

9 $$CDX_{i,t + h} = \alpha_{i} + \beta_{i,1} CDX_{i,t} + \gamma_{i}^{F} F_{t} + \user2{ }\varepsilon_{i,t + h} .$$

Next, we augment the predictive model in Eq. (9) by adding one-by-one macroeconomic indicators:

10 $$CDX_{i,t + h} = \alpha_{i} + \beta_{i} CDX_{i,t} + \gamma_{i}^{F} F_{t} + {\varvec{\gamma}}_{{\varvec{i}}}^{{\varvec{M}}} {\varvec{M}}_{{\varvec{t}}} + \varepsilon_{i,t + h}$$

and measures of economic uncertainty, leading to the predictive model:

11 $$CDX_{i,t + h} = \alpha_{i} + \beta_{i} CDX_{i,t} + \gamma_{i}^{F} F_{t} + \gamma_{i}^{M} M_{t} + {\varvec{\gamma}}_{{\varvec{i}}}^{{\varvec{U}}} {\varvec{U}}_{{\varvec{t}}} + \varepsilon_{i,t + h}$$

In line with *Hypothesis **4*, we set the predictive horizon at *h* = *3* months ahead.^19^

We evaluate the predictive ability by utilizing the mean squared error (MSE) statistic that measures the expected value of the quadratic loss. To assess significance, we employ the Clark and West (2007) one-sided MSE-adjusted t-test. The test statistic indicates whether an extended model generates superior OOS forecasts than a simpler, nested model. A positive test statistic indicates that an extended model produces more accurate forecasts than the nested model whereas the negative test statistic signals that an extended model contains unnecessary predictive variables that introduce noise to the forecast MSE. Table 7 reports the results.

**Table 7:** Predictive ability of macroeconomic variables within a static predictive model

	Panel A: CDXIG spread					Panel B: CDXHY spread				
Model	RMSE	RMSE ratio	CW statistic	RMSE ratio	CW statistic	RMSE	RMSE ratio	CW statistic	RMSE ratio	CW statistic
Model Extention										
Baseline 1	13.16					57.84				
*Extentions with macroeconomic indicators*										
IP		0.99	1.04	0.99	1.04		1.01	− 0.09		
PMI		0.74	2.79***	0.74	2.79***		0.85	3.04***	0.88	2.28**
UG		1.01	1.57*	1.01	1.57*		1.02	1.14	1.02	0.82
NFP		0.99	1.67**	0.99	1.67**		1.01	-0.40		
CPI		1.21	0.44				1.32	− 0.05		
*Extentions with uncertainty measures*										
IPSD		1.17	− 2.19				1.17	− 1.51		
PMISD		1.02	− 1.13				1.02	− 0.52		
URSD		0.99	0.67				0.99	0.77		
NFPSD		0.97	1.71**	0.95	1.75**		0.97	2.13**	0.92	1.93**
CPISD		1.06	− 0.84				1.05	− 0.61		

The first row in each panel reports the RMSE of the Baseline 1 CDX spread model, Eq. (6). The subsequent rows across each panel list variables added to the model in the preceding row, followed by the ratio of forecast RMSEs of the model at hand and the nested model from the preceding row. A ratio RMSE < 1 indicates that the additional macroeconomic predictor in the extended model brings a forecast error reduction vis-à-vis the nested model. Significance of the forecast MSE differential is tested with the Clark and West (2007); CW t-statistic for the null hypothesis that the predictive ability of the extended model is not superior to that of the nearest nested model. *, **, and *** denote rejection at the 10%, 5% or 1% level, respectively. The second set of results in the last two columns of each panel gives the RMSE ratio and the CW test statistic for extensions of the Baseline 1 model, Eq. (6), that employ informative predictors only, i.e. those that at least marginally reduce the mean forecast error according to the CW test statistic reported in the preceding column. Estimation is based on monthly data and the forecast horizon is h = 3 months (quarter ahead)

### Forecasting results

The results in Panel A, Table 7 indicate that for CDXIG, successive *Baseline 1* extensions by PMI, UG, and NFP, Eq. (7), all deliver significant improvements in the prediction accuracy, over and beyond that of the *Baseline 1* model, Eq. (6), with IP, albeit marginally, also contributing to a reduction in the forecast RMSE. Similarly, for CDXHY in Panel B, PMI and UG help improve the forecast RMSE. Among measures of macroeconomic uncertainty, the results in Panel A and Panel B for Eq. (8) highlight NFPSD as an important predictor for both CDXIG and CDXHY.

To reduce the effect of noise from uninformative predictors on the forecast RMSE, we repeat the forecasting exercise, this time retaining only those macroeconomic predictors that have delivered at least a marginally significant RMSE reduction as indicated by the CW test statistic; the last two columns in Panels A and B, Table 7 report the results. The results indicate that the baseline model extension with relevant macroeconomic indicators and uncertainty measures, Eq. (8), deliver a sizable improvement in the prediction accuracy: the total reduction in the forecast RMSE relative to the *Baseline 1* model, Eq. (6), $\left(1-\frac{{RMSE}_{Eq. (8)}}{{RMSE}_{Eq. (6)}}\right)$ is 30.9% and 17.1%, respectively, for CDXIG and CDXHY. Macroeconomic indicators jointly deliver most of this reduction, $\left(1-\frac{{RMSE}_{Eq. (7)}}{{RMSE}_{Eq. (6)}}\right)$, at 27.3% for CDXIG and 9.9% for CDXHY, with PMI showing by far the largest contribution for both CDX indices. Nevertheless, the contribution of uncertainty measures $\left(1-\frac{{RMSE}_{Eq. (8)}}{{RMSE}_{Eq. (7)}}\right)$, is also notable, particularly at 8.0% for CDXHY, being only slightly lower than the 9.9% contribution of macroeconomic indicators.

Table 8 reports the forecasting results for the dynamic model specification, Eqs. (9) - (11). For Eq. (9), the results suggest that augmenting *Baseline 1* with the AR(1) term leads to a notable improvement in the forecasting accuracy of the resulting model, *Baseline 2*, achieving the RMSE reduction $\left(1-\frac{{RMSE}_{Eq. (9)}}{{RMSE}_{Eq. (6)}}\right)$ of 24.6% for CDXIG and 22.6% for CDXHY.

**Table 8:** Predictive ability of macroeconomic variables within a dynamic predictive model

	Panel A: CDXIG spread					Panel B: CDXHY spread				
Model	RMSE	RMSE ratio	CW statistic	RMSE ratio	CW statistic	RMSE	RMSE ratio	CW statistic	RMSE ratio	CW statistic
Model Extention										
Baseline 2	9.92					44.75				
*Extentions with macroeconomic indicators*										
IP		1.01	− 0.12	1.01	− 0.12		1.06	− 0.15		
PMI		0.87	2.49***	0.87	2.49***		0.91	3.05***	0.95	2.14**
UG		1.02	1.18	1.02	1.18		1.01	1.20	1.02	0.73
NFP		1.02	− 0.03	1.02	− 0.03		1.05	0.28		
CPI		1.20	− 1.43				1.35	− 1.36		
*Extentions with uncertainty measures*										
IPSD		1.08	− 1.58				1.06	− 1.34		
PMISD		1.03	− 0.80				1.02	− 0.19		
URSD		1.01	− 0.60				1.01	− 0.42		
NFPSD		0.99	1.33*	0.98	1.52*		0.99	1.38*	0.97	1.94**
CPISD		1.07	− 1.03				1.06	− 0.86		

The first row in each panel reports the RMSE of the Baseline 2 CDX spread model, Eq. (9). The subsequent rows across each panel list variables added to the model in the preceding row, followed by the ratio of the forecast RMSEs of the model at hand and the preceding (nested) model. A ratio RMSE < 1 indicates that the additional macroeconomic predictor in the extended model brings a forecast error reduction vis-à-vis the nearest nested model from the preceding row. Significance of the forecast MSE differential is tested with the Clark and West (2007); CW t-statistic for the null hypothesis that the predictive ability of the extended model is not superior to that of the nested model. *, **, and *** denote rejection at the 10%, 5% or 1% level, respectively. The second set of results in the last two columns of each panel report the forecast RMSE ratio and the CW test statistic for extensions of Baseline 2 model with informative predictors only, identified in Table 7. Estimation is based on monthly data and the forecast horizon is h = 3 months (quarter ahead)

For Eqs. (10) and (11), as before, we begin the analysis by employing a full set of candidate predictors and then repeat the analysis using only a sub-set of informative predictors identified in Table 7. Notably, despite the AR(1) term absorbing much of the predictive power of the conventional determinants, for CDXIG and CDXHY, both, PMI and NFPSD retain their predictive ability, delivering a significant improvement to the forecast RMSE.^20^ Using the sub-set of informative predictors only, the macroeconomic predictors jointly reduce the forecast RMSE, $\left(1-\frac{{RMSE}_{Eq. (11)}}{{RMSE}_{Eq. (9)}}\right),$ by 10.9% and 6.1% for CDXIG and CDXHY, respectively. The relative contribution of macroeconomic indicators as a group remains notably higher than that of uncertainty measures for CDXIG. For CDXHY, the 3.0% RMSE reduction delivered by the uncertainty measures is comparable to that achieved by the macroeconomic indicators.

Our findings of the useful predictive ability of macroeconomic variables for CDX spreads, beyond that of the conventional theoretical determinants, within both static and dynamic specifications of the predictive model, provide clear evidence against *Hypothesis **4*.

Our analysis provides novel evidence on the CDX spreads predictability in real time. As a by-product of our analysis, we identify PMI as a variable with the strongest predictive ability for a-quarter-ahead CDX spreads. Along with the NFP-related uncertainty measure, PMI notably improves real-time spread forecasts delivered by the conventional predictors. Our CDS market related evidence complements findings on the predictive ability of macroeconomic variables in the context of U.S. Treasuries, for example, in Ang and Piazzesi (2003) and Ludvigson and Ng (2009), and equity markets in Rapach et al. (2010) and Paye (2012).

Fama and French (1989) and Cochrane (1999, 2007) provide intuition relating to risky assets, also of relevance here, by pointing out that their market risk premium fluctuates with the business cycle, rising in downturns, driven by elevated investor risk aversion, thus generating the market risk premium predictability. This intuition is relevant for CDS as both default risk and risk premium components of the CDS spread are known to strongly vary with the business cycle (e.g., Berndt et al. 2018, and Yfanti et al. 2023). Rapach et al. (2010) further show that the macroeconomic variables, which they find contain predictive information for equity risk premium, can also predict business cycles, thereby corroborating the argument in Cochrane (2007) that the predictability of financial asset prices is related to macroeconomic risk. Our finding of the predictive content for CDX in PMI and NFPSD, both representing *forward-looking* survey-based macroeconomic measures, is consistent with this argument.

Taken together, the arguments put forward by these authors also help reconcile our evidence with findings in a parallel strand of literature which suggests that some market-based measures relating to equity premium and bond term spread can forecast real output growth and recessions as, for example, in Harvey (1989), Estrella and Hardouvelis (1991) and Ang et al. (2006), by pointing out that the predictive content in these market variables is linked to their ability to capture forward-looking macroeconomic information. Accordingly, the predictability of both financial market and macroeconomic measures in the two strands of literature is linked to macroeconomic risk and the ability of the predictors at hand to capture forward-looking data about the economic environment.

Furthermore, in two recent studies, Kiesel et al. (2016) and Kiesel et al. (2021) document delays in CDS spreads reflecting new complex information, in their case relating to credit rating announcements, thereby highlighting another factor that can play a role in CDS predictability. The evidence therein suggests that even informed active market participants can face some initial uncertainty about the impact of complex macroeconomic changes on CDS, leading to a delay in fully embedding this information into CDS spreads.^21^ However, Kiesel et al. (2021) argue that such delay does not necessarily signal market inefficiency, with further research needed to establish whether it can be exploited for generating abnormal profits. The gradual processing of macroeconomic news by market participants, as they place together pieces of incoming information and update their view about the economy, is also broadly consistent with the theoretical argument in David (2008) and David and Veronesi (2013), emphasizing learning effects linked to investors’ uncertainty about the state of the economy.

## Conclusions

Motivated by a high level of economic uncertainty that can persist in real time, affecting firms’ credit risk and its pricing by market participants, we examine the informational content in various macroeconomic indicators and associated real-time uncertainty measures for investment-grade and high-yield CDX index spreads. Our analysis focuses on the post-GFC economic recovery period that saw a rapid increase in activity in CDS index instruments as market participants sought economy or sector-wide credit risk protection.

Our analysis identifies measures of economic output, employment, inflation, and labor market-related uncertainty that provide significant explanatory power for investment-grade and high-yield CDX spreads, influencing CDX beyond the impact of the conventional theoretical determinants. This finding helps address the credit spread puzzle and provides indirect evidence that market participants likely use multiple economic measures in real time for gauging the economy in their CDS pricing. Our analysis further reveals that investment-grade and high-yield CDX differ in their sensitivity to various economic aspects such as output, labor market conditions, and associated uncertainty, thereby providing an explanation for some conflicting findings in the previous literature. Also, our evidence indirectly corroborates the findings in Amato and Luisi (2006) that the informational content in inflation measures can be limited during periods when inflation is low and stable.

Taken together, these findings emphasize the importance of accurately capturing the key aspects of the macroeconomy in modeling and analyzing CDS spreads. The findings argue against using either only economic output measures or broad data aggregation across economic aspects as routinely done in the CDS literature. Our evidence also informs the growing literature on the term structure modeling of credit spreads. This literature has grouped economic output and employment variables together under the “real activity” heading, analyzing the impact of an aggregate measure as in Amato and Luisi (2006), Wu and Zhang (2008), and Zhou (2014), or considered only economic output variables while overlooking the employment aspect altogether as in Yang (2008) and Dewachter et al. (2019).

Our evidence further suggests that macroeconomic risk pricing embedded in CDX spreads differs for investment and speculative-grade borrowers. Consequently, using the market risk premium associated with investment-grade entities to analyze the impact of the macroeconomy, as routinely done, for both high-yield and investment-grade CDS can lead to the underestimation of its impact for high-yield CDS. This finding has implications for CDS studies that employ market-based measures of the macroeconomy.

Our OOS predictive analysis provides further evidence of significant predictive content in some macroeconomic variables for a-quarter-ahead CDX spreads, over and beyond the predictive content in the conventional predictors alone. This finding complements the related evidence in the context of equity markets in Rapach et al. (2010) and Paye (2012) and U.S. Treasuries in Ang and Piazzesi (2003) and Ludvigson and Ng (2009) inter alia. Furthermore, the evidence of CDX predictability is of importance to market participants for reducing the cost of credit risk hedging. Investors can also benefit by exploiting a strong correlation of CDX spreads with macroeconomic variables to better diversify their portfolios.

These empirical findings also motivate the need for future work in the emerging area of the informational efficiency in the CDS market context, focusing on the informational content of CDS spreads in relation to uncertainty about credit risk linked to macroeconomic conditions. This new direction complements the recent related work in Kiesel et al. (2021), focusing on uncertainty in credit risk associated with credit rating reviews, as well as the earlier work of Jenkins et al. (2016) on the informational efficiency of the CDS market with regards to post-earnings announcement returns.

## Data Availability

No original data was collected or generated during this study.

## Footnotes

1 ISDA (2019) reports the quarterly MRTA figures using a four-quarter rolling average basis.

2 Another important feature that makes CDS indices particularly appealing to investors is that the CDS index contract does not terminate if a member of the CDS index portfolio defaults as is the case for a single-name CDS or a basket CDS, continuing trading with a reduced notional amount (Alexander and Kaeck 2008).

3 Tang and Yan (2010) also include investor sentiment.

4 The start of our sample period coincides with the beginning of the expansion period following the GFC as identified by the National Bureau of Economic Research; see https://www.nber.org/research/data/us-business-cycle-expansions-and-contractions.

5 Market-based measures in Chan and Marsden (2014), constructed using daily financial and housing market data, are also subject to noisy data-related limitations (e.g., Claessens and Kose 2017).

6 Chan and Marsden (2014) and Cao et al. (2022) also examine, respectively, a-day-ahead and a-month-ahead predictability of CDS spread changes. However, in contrast to Narayan et al. (2014), Avino and Nneji (2014), and our study, focusing on the predictability out-of-sample, or in real time, their scope is limited to the in-sample analysis. However, the in-sample predictability does not directly translate into predictability in real time. Also, the prediction horizon in both studies is shorter than ours.

7 Surprisingly, Chan and Marsden (2014) document a positive association between their measure of business climate and CDXHY during tranquil periods which also contradicts the findings in Kim et al. (2017).

8 Campello et al. (2010) document that the unavailability of external financing in 2008 resulted in low-rated firms selling their assets and burning through internal cash reserves to fund their operations during the crisis while high-rated firms’ assets and cash reserves remained relatively intact.

9 Collin-Dufresne et al. (2001) is another study of theoretical determinants of corporate credit spreads that employs a linear regression analysis.

10 For consistency, we transform economist nowcasts of the monthly change in the total nonfarm payroll employment into the monthly percentage change by dividing by the total nonfarm payroll in the previous month, before calculating the NFPSD and NFPIQR measures.

11 The CDXIG and CDXHY indices are owned and managed by IHS Markit.

12 The nature of the relationship of UG, NFP, and the uncertainty measures with CDX is difficult to discern from the graphical representation; hence, it is not shown here.

13 ${EV}_{t}$ is employed after eliminating common variation in ${EV}_{t}$ and ${ER}_{t}$ as the two variables exhibit notable correlation. We regress ${EV}_{t}$ on ${ER}_{t}$, using the regression residual in the analysis.

14 We employ the Newey-West estimator with one lag using a Barlett kernel. Following Andrews and Monahan (1992) and Newey and West (1994), we apply a pre-whitening step, with the lag order selected using the Schwarz information criterion.

15 We further note that IP becomes insignificant for CDXIG when the conventional regressors capturing the risk-free rate, asset value growth, and volatility are added to the regression in Eqs. (4) and (5), reported in Table 5.

16 Our sensitivity analysis is primarily based on Eqs. (1) and (2) as the results relating to augmented Eqs. (4) and (5) can produce biased interpretations of the impact of individual macroeconomic variables due to additional conventional regressors in the latter equations capturing some of the macroeconomic influences.

17 Hatzius and Stehn (2018) highlight an additional complication arising from utilizing various financial conditions indices such as those produced by Bloomberg, the IMF, the Chicago Fed, and Goldman Sachs, with the latter employed in Kim et al. (2017), that while their trajectories tend to be similar during crisis times, they differ notably during normal times.

18 To gauge the usefulness of the predictive content of the AR(1) term, we compare forecasts generated by a univariate AR(1) model to forecasts produced by a random walk (RW) model, a widely used benchmark. As expected, the AR(1) model generates competitive forecasts relative to RW, with the respective forecast RMSE of 9.63 vs 10.08 for CDXIG and 43.10 vs 45.30 for CDXHY.

19 For the OOS forecasting analysis, we split our data observations using the 0.6:0.4 ratio to obtain the initial estimation window and the hold-out window. The forecasts are obtained recursively. As a robustness check, we repeated the predictive analysis using the 2/3:1/3 ratio for splitting the data points; the results (not reported here for brevity) are available upon request. The two sets of forecasting results are very similar and support our analysis.

20 In a separate exercise, we find that in *Baseline 2*, the AR(1) term delivers most of the predictive power. In contrast, the conventional theoretical determinants contribute very little to the model’s predictive ability, with only RF delivering a significant RMSE reduction for CDXIG (but not CDXHY). The details, not shown here for brevity, are available upon request.

21 The complexity arises due to macroeconomic changes affecting CDS not only directly via the market price of risk but also indirectly, through modifying firms’ financing and other business policies that, in turn, impact their future risk of default.
